# Age-related changes in the spatiotemporal responses to electrical stimulation in the visual cortex of rats with progressive vision loss

**DOI:** 10.1038/s41598-017-14303-1

**Published:** 2017-10-26

**Authors:** Soshi Miyamoto, Naofumi Suematsu, Yuichi Umehira, Yuki Hayashida, Tetsuya Yagi

**Affiliations:** Biosystems and Devices Area, Division of Electrical, Electronic and Information Engineering, Department of Electronic Engineering, Graduate School of Engineering, Osaka University, 2-1 Yamadaoka, Suita, Osaka 565-0871 Japan

## Abstract

The Royal College of Surgeons (RCS) rat gradually loses vision due to retinal degeneration. Previous physiological studies have depicted the progressive loss of optical responses in the visual pathway, including the primary visual cortex (V1), over the course of retinal degeneration in the RCS rat. However, little is known about how the excitability of the V1 circuit changes during over the course of the gradual loss of visual signal input from the retina. We elucidated the properties of responses to electrical stimulations directly applied to V1 at different stages of vision input loss in the RCS rat in reference to those of the Long-Evans (LE) rat, using *in vivo* voltage-sensitive dye imaging. The V1 neuronal network of the RCS rat exhibited an excitatory response comparable to the LE rat. The excitatory response was maintained even long after total loss of the visual signal input from the retina. However, the response time-course suggested that the suppressive response was somewhat debilitated in the RCS rat. This is the first experiment demonstrating the long-term effect of retinal degeneration on cortical activities. Our findings provide the physiological fundamentals to enhance the preclinical research of cortical prostheses with the use of the RCS rat.

## Introduction

The Royal College of Surgeons (RCS) rat gradually loses vision due to retinal degeneration initiated by deterioration of the photoreceptor layer^1–4^. Previous physiological studies have depicted the progressive loss of optical responses in the visual neuronal networks over the course of the retinal degeneration in the RCS rat. Electrophysiological measurements using electroretinograms (ERGs) showed that the response to illumination in RCS rats was maintained at a similar level to that of congenic non-dystrophic rats for the first 27 days, which gradually diminished and then almost disappeared by postnatal day 101^5^. In parallel to the diminishing response amplitude, the latency of the response was prominently prolonged^6,7^. Similar degradation of the light-induced response was reported in the following visual pathway, i.e., the superior colliculus (SC)^4,8^ and the primary visual cortex (V1)^3^, over the course of gradual retinal degeneration. The multi-unit activity (MUA) that was recorded in the SC of the RCS rat showed a gradual decrease in activity and an increase in latency of response to optical stimulation^4,8^. The local field potential (LFP) and MUA, as well as intrinsic signals recorded in response to the optical stimulation in V1, also degraded with a similar time-course^3^. These studies clearly demonstrate the impact of retinal degeneration on the response properties of the post-retinal visual processing areas. However, degradation of the light-induced response in the post-retinal visual areas can partially be attributed to activity changes of the post-retinal visual areas themselves under the influence of a diminishing or absent visual input signal.

Since the pathology of progressive vision loss is similar to that of degenerative retinal diseases in humans, e.g., retinitis pigmentosa, the RCS rat is considered to be a suitable animal model for preclinical studies to develop new therapies for acquired vision loss^9–16^.

Cortical visual prosthetics represent one of these possible therapies to restore partial vision in blind patients^17–21^. In cortical visual prosthetics, electrical stimulations encoding the incoming image are delivered with multichannel electrodes directly onto the visual cortex of the blind patient to evoke a phosphene image consisting of spot-like light sensations. Cortical prosthetics can be broadly applied to blindness due to the dysfunction of subcortical visual pathways^22–25^, provided that the cortical circuit maintains its responsiveness to electrical stimuli after the absence of visual input from the retina. The RCS rat can provide enlightening opportunities for preclinical experiments in cortical prosthetic research. For example, behavioural experiments can be designed by associating light sensations with electrical stimulations, because of the slow and gradual loss of the visual function. It is also possible to study remodelling events induced by electrical stimulations onto the cortical circuits. In this regard, it is important to measure the response properties of the visual cortex in the RCS rat and to verify if the excitability of the cortical neuronal network changes over the course of gradual retinal degeneration.

In the present study, we elucidated the neuronal response properties to optical and electrical stimulations in V1 at different stages of vision loss in the RCS rat in reference to those in the Long-Evans (LE) rat. To measure the spatiotemporal properties of the optically and electrically induced responses, we conducted *in vivo* imaging with voltage-sensitive dye (VSD), which is known to probe population postsynaptic potentials (PSPs)^26–28^. We demonstrate that the V1 neuronal network in the RCS rat exhibited an excitatory response to directly applied electrical stimulations that is comparable to the response in the LE rat. Excitability was maintained through the *end*-stages of retinal degeneration, even to the period long after the total loss of visual signal input from the retina. However, the recovery phase of the electrically induced response in the RCS rat was significantly slower than that of the LE rat, which suggests that the suppressive response was somewhat debilitated in the RCS rat. This is the first experiment studying the long-term effect of retinal degeneration on cortical activities, and our findings provide the physiological fundamentals to enhance the preclinical research of cortical prosthetic devices with the use of the RCS rat.

## Results

### Light-induced cortical responses

**Figure 1:**
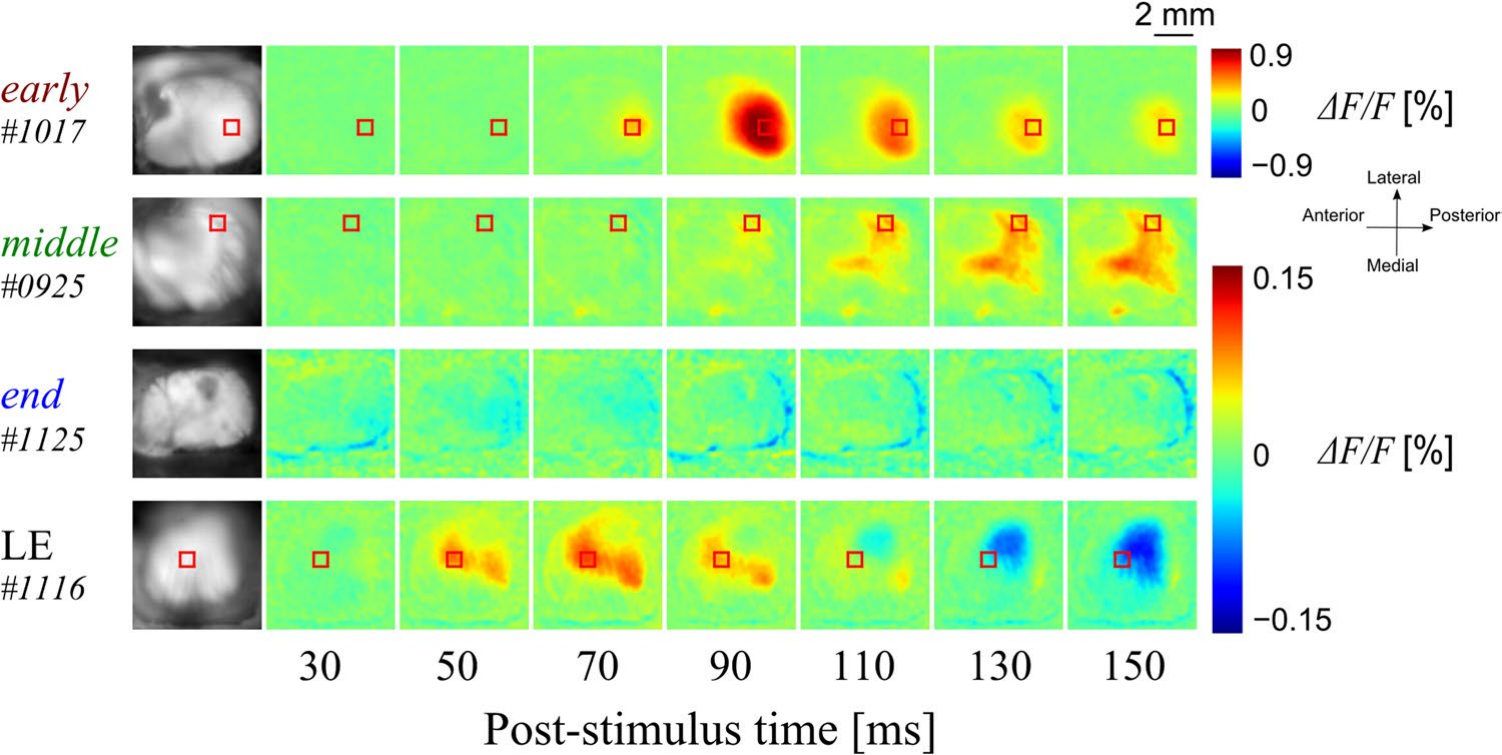
Typical *ΔF/F* image sequences indicating the light-evoked responses in *early-* (3-week-old), *middle-* (10-week-old), and *end-* (99-week-old) stage RCS rats, and LE (9-week-old) rats. Green, red, and blue colours indicate no change, an increase, and a decrease of neural cortical activities compared to pre-stimulation levels, respectively. The leftmost column is the VSD fluorescent image showing the stained area in white. The red rectangles indicate the averaged regions (11 × 11 pixels), which were used for the time-courses (see Methods). The *middle*-stage RCS rats had a longer response latency than the *early*-stage rats, and no light-evoked response was observed in the *end*-stage RCS rats. The LE rats had the shortest response latency in these examples.

To study the properties of light-induced cortical responses in the RCS and LE rats, we presented flashing-light stimuli to one eye and measured VSD images in the contralateral visual cortex. Figure 1 shows representative examples of the cortical neuronal responses to light stimulation in each group. As illustrated in Fig. 1, the response appeared in the visual cortex within 50 ms in the LE rat. The LE rat exhibited a late-phase (110 ms and later frames) undershoot response, i.e., the signal was lower than that in the pre-stimulation period (shown in blue). However, in the RCS rats at the *early* and *middle* stages of degeneration, the response was not discernible at 50 ms after the onset of the stimulus. The excitatory response (positive deflection of the VSD signal) appeared at 70 ms following the onset of the stimulus and remained even after 150 ms. No undershoot was seen until after 150 ms in these animals. The representative RCS rat at the *end* stage of degeneration did not respond to the light stimulus at all, suggesting that there is no input from the retina to the visual cortex at this *end* stage of disease^2,10,29^.

**Figure 2:**
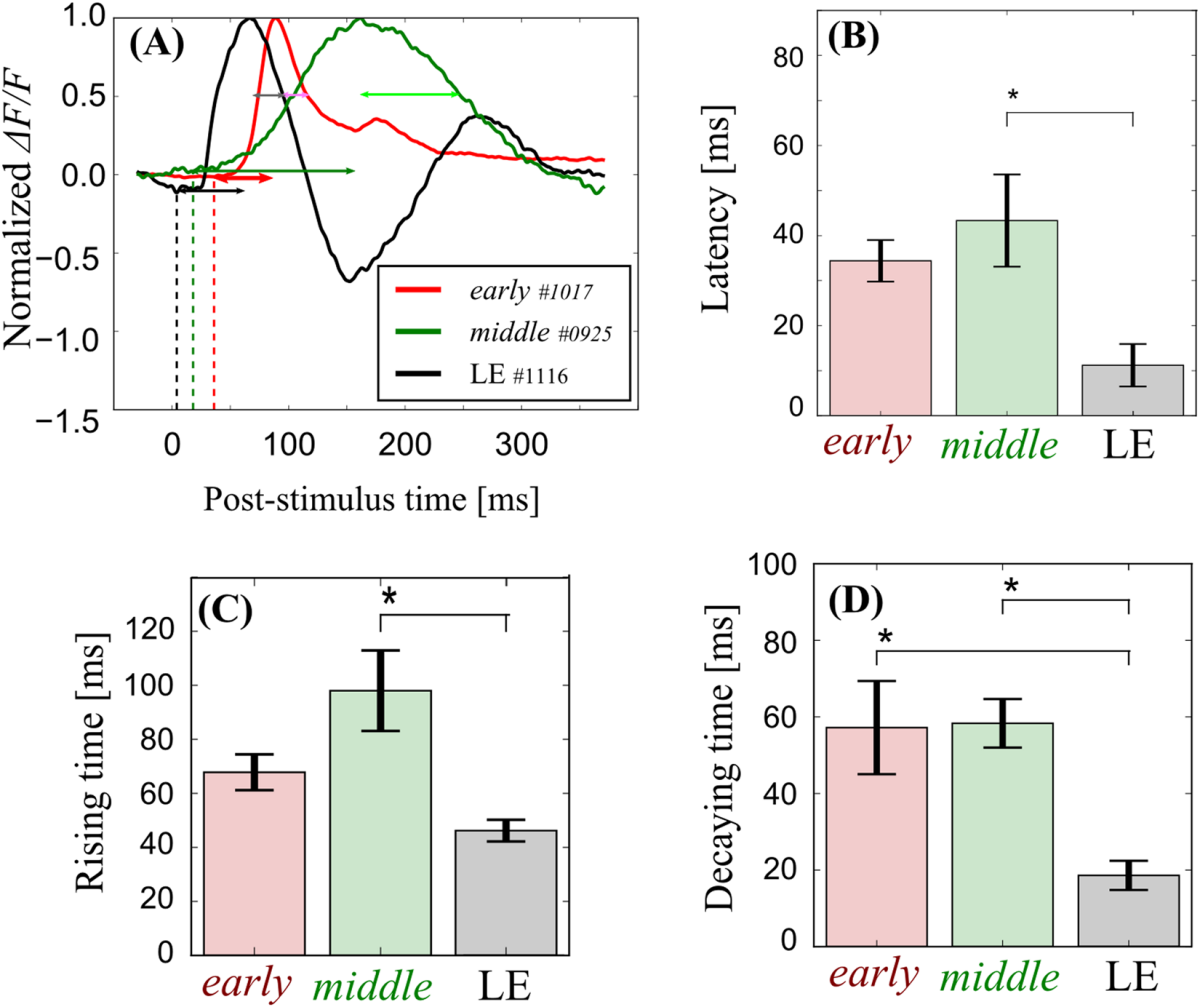
The temporal dynamics of light-evoked cortical responses. (**A**) The normalized time-courses of the typical examples indicated in Fig. 1 [red, *early* (3 weeks old); green, *middle* (10 weeks old); black, LE (9 weeks old)]. The vertical dashed lines indicate the response latency. The dark and bright horizontal arrows indicate the rising time and decaying time, respectively. Note that the *end*-stage RCS rat data were omitted since no obvious responses were evoked by the flashing-light stimulation. (**B**–**D**) Comparison of the response latency, rising time, and decaying time between all of the groups (mean ± SEM, *p < 0.05). All the temporal parameters in the RCS rats, especially older ones, tended to be longer.

Figure 2 (A) depicts the time-course of the response of a representative animal from each group, except for the *end*-stage RCS rats. The difference in the response times between the LE rat and the RCS rats is clearly visible. The response initiated at 4 ms and peaked at 66 ms in the LE rat. The response amplitude quickly decreased and a distinct undershoot response was observed. A similar undershoot response was seen in all LE rats (*n* = 5). However, the RCS rats showed longer response latency (36 and 18 ms for *early*- and *middle*-stage rats, respectively). The response peak also delayed in the RCS rats compared to the LE rat. No undershoot response was seen in these *early*- and *middle*-stage RCS rats. Although the undershoot was seen in one out of five *early*-stage RCS rats, and two out of six *middle*-stage RCS rats, the time to the undershoot peak from the onset of the stimulus was much longer (>250 ms) than that of the LE rats.

Figure 2(B), (C) and (D) show the statistical analyses of the response latency, the rising time and the decaying time (see Methods), respectively. As shown in Fig. 2(B), the average response latency was shorter in the LE rats compared to the RCS rats. The average response latency became longer as the degeneration progressed in the RCS rats [one-way ANOVA, *F*_(2,13)_ = 27.8, *p* = 2.03 × 10^−5^]. There was a statistically significant difference between the *middle*-stage RCS rat and the LE rats [post hoc Tukey-Kramer method, *q*_(3,13)_ = 10.4, *p* = 0.001]. To evaluate the speed of the rising phase and the falling phase, the rising time and the decaying time are compared in Fig. 2(C) and (D), respectively. Again, the rising time became longer as the degeneration progressed in the RCS rats [*F*_(2,13)_ = 5.11, *p* = 0.023]. The rising time was significantly longer in the *middle*-stage RCS rats than the LE rats [*q*_(3,13)_ = 4.45, *p* = 0.019]. The decaying time was shorter in the LE rats than in the *early*-stage RCS rats [*F*_(2,13)_ = 6.23, *p* = 0.013; *q*_(3,13)_ = 4.19, *p* = 0.028] and in the *middle*-stage RCS rats [*q*_(3,13)_ = 4.50, *p* = 0.018]. The peak-to-trough (see Methods) time was also assessed for the data that displayed an undershoot. The *early*- and *middle*-stage RCS rats and the LE rats had peak-to-trough values of 179 ± 21.9, 177 ± 8.08, and 94 ± 8.09 ms, respectively [mean ± standard error of the mean (SEM); *n* = 3, 5, and 4 for the *early*- and *middle*-stage RCS rats and the LE rats, respectively; *F*_(2,13)_ = 14.3, *p* = 0.002; *early* vs. LE, *q*_(3,13)_ = 6.28, *p* = 0.005; *middle* vs. LE *q*_(3,13)_ = 6.63, *p* = 0.004]. These observations strongly suggest that the light-induced response becomes slower as degeneration progresses in the RCS rat.

### Temporal properties of the electrically induced cortical response

In the previous section, we showed that the light-induced cortical response becomes slower with age, and finally disappears in the V1 of the RCS rat. This gradual change in the light-induced response may be attributed to changes in the cortical circuit itself or in the subcortical visual pathway, such as the LGN and the retina. In this section, we examined whether the cortical circuit changes its responsiveness to electrical stimuli during the gradual loss of retinal input.

**Figure 3:**
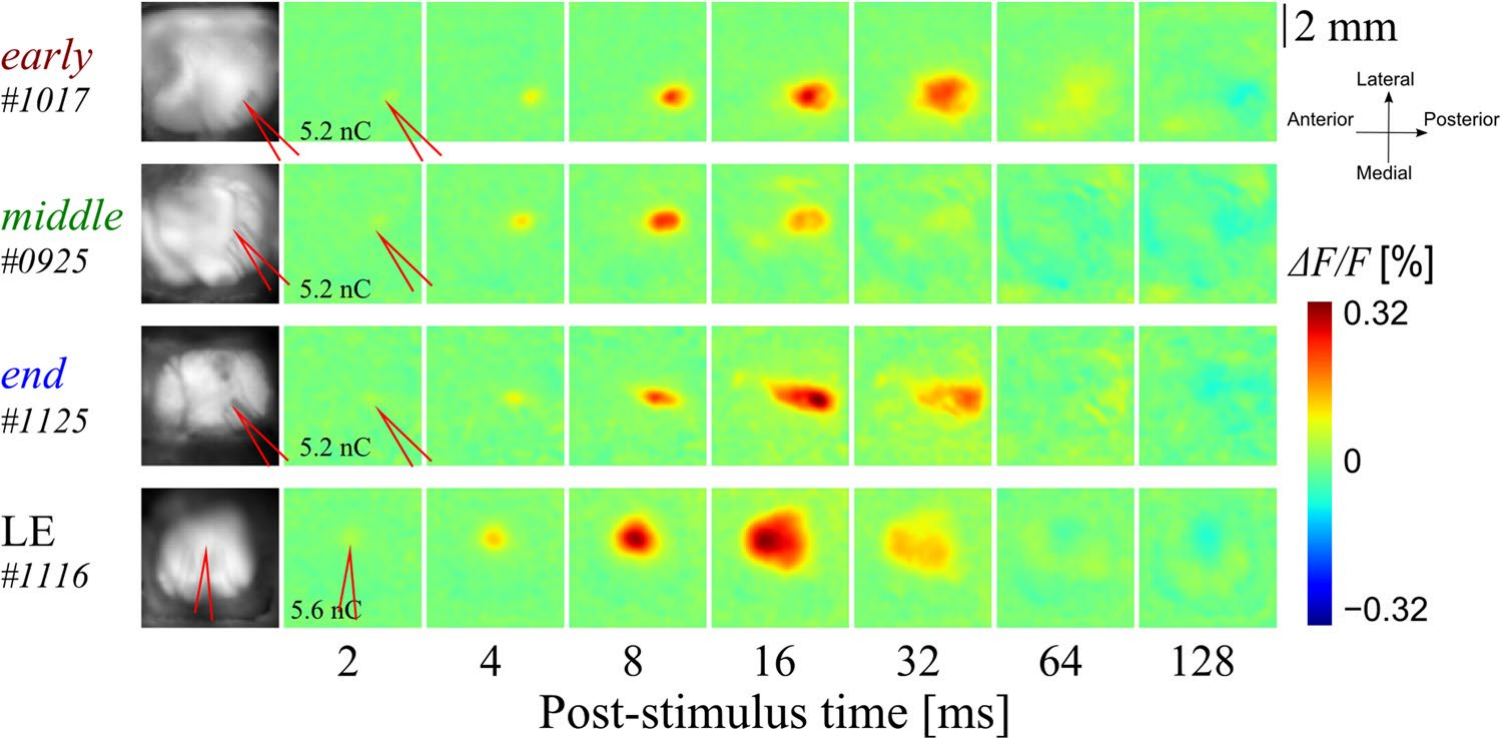
Typical examples of *ΔF/F* image sequences showing the responses to electrical stimuli with the same rat indicated in Fig. 1. The stimuli were applied through electrodes inserted into the V1 area (red lines in the leftmost and second leftmost columns indicate the tips of the electrodes). The values of the second column from the left indicate the stimulus intensity. For a better view of the response rising phases, we used log-spaced timepoints. Similar responses were induced in all rats, and neural excitation was even induced in the *end-*stage RCS rats, although no light-evoked response was found.

Figure 3 shows representative examples of electrically induced neuronal activity. In contrast to the light-induced responses (Figs 1 and 2), similar time-courses were recorded in the rising phase among all rats, including the LE rat. The responses to electrical stimuli around the stimulation site (see Methods) appeared at 2–4 ms and peaked at approximately 7 ms after the stimulation in all rats. Subsequently, the responding areas expanded. This observation indicates a lateral spread of excitation, as was seen previously in the mouse^30^ and rat^31^.

**Figure 4:**
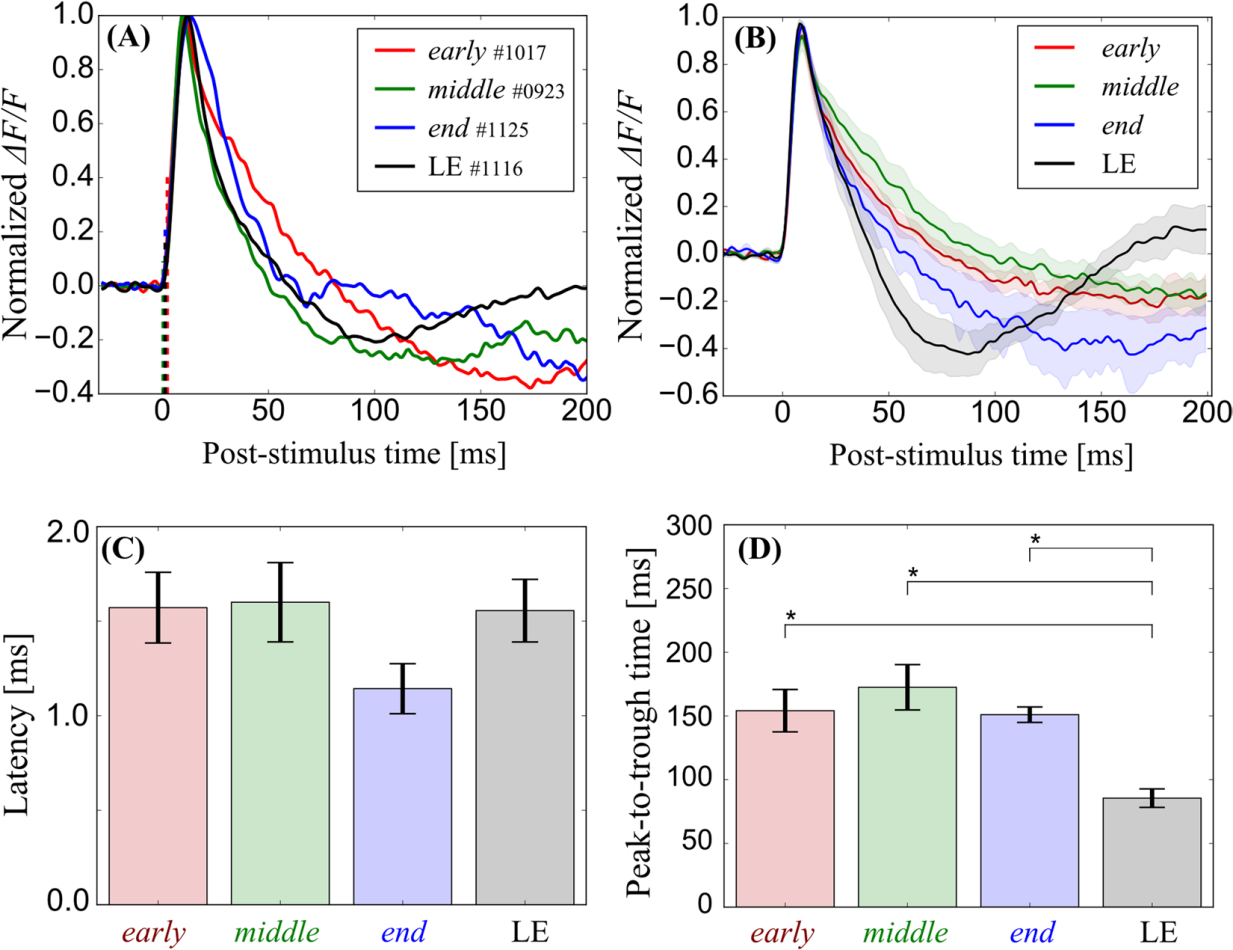
The temporal properties of cortical responses to the electrical stimuli. (**A**) Typical examples of the normalized time-courses at the stimulation point indicated in Fig. 3. Red, green, blue, and black lines indicate the time-courses of the *early-* (3-week-old), *middle-* (10-week-old), and *end-* (99-week-old) stage RCS rats, and the LE rat, respectively. The vertical dashed lines indicate the response latency. (**B**) The averaged time-course for each group (mean ± SEM). The response latencies appeared to be similar in all groups. On the other hand, the response durations appeared to be different between the RCS rat groups and the LE rat group. (**C**,**D**) Comparisons of the latency and the peak-to-trough time (mean ± SEM, *p < 0.05). The RCS rat groups exhibited longer peak-to-trough time.

The time-course of the responses in Fig. 3 is depicted in Fig. 4(A). In these animals, the response latency was 2, 1 and 2 ms for the representative *early*-, *middle*- and *end*-stage RCS rats, respectively (vertical red, green, blue dashed lines, respectively). The response latency in the representative LE rat was also 2 ms (vertical black dashed line). Figure 4(B) shows normalized and averaged response time-courses around the stimulation point for each group. As shown in the examples, the electrically induced cortical responses exhibited similar rising but different falling time-courses between the LE rats and the RCS rats.

The average latencies were 1.6, 1.6, and 1.1 ms in the *early*-, *middle*- and *end*-stage RCS rats, respectively [Fig. 4(C)]. The average latency was 1.6 ms in the LE rats. No significant difference was observed between all groups [*F*_(3, 25)_ = 1.10, *p* = 0.364]. The differences between all rat groups were within a time resolution of the VSD signal measurement, suggesting that the excitability in the cortical circuit is maintained even after the total loss of retinal input in the RCS rat.

The difference in the falling time-course between the LE and RCS rats is thought to be relevant to the undershoot response. The time-course of the undershoot is similar to inhibitory PSPs (IPSPs) electrically induced in the visual cortex in slice preparations^32^, as well as *in vivo* ^30^ in rodents. In the LE rats, eight out of nine animals exhibited an undershoot response that had a negative amplitude peak. The undershoot response was also seen in 21 out of 24 RCS rats. However, the time-course of these undershoot responses was different between the RCS and LE rats. To quantify the difference, the peak-to-trough time was measured and is shown in Fig. 4(D). The peak-to-trough time was approximately 150 ms in the RCS rats at all disease stages. The peak-to-trough time of the LE rats was approximately 90 ms. In contrast to the latency, the peak-to-trough time showed a significant difference between the RCS rats and the LE rats [*F*_(3, 25)_ = 7.43, *p* = 0.001; *early* vs. LE*, q*_(4, 25)_ = 4.78, *p* = 0.011; *middle* vs. LE, *q*_(4, 25)_ = 6.27, *p* = 0.001; *end* vs. LE, *q*_(4, 25)_ = 4.37, *p* = 0.023].

These observations could be explained by decreased suppressive activity in RCS rats compared to LE rats (see Discussion).

### Current intensity dependence

**Figure 5:**
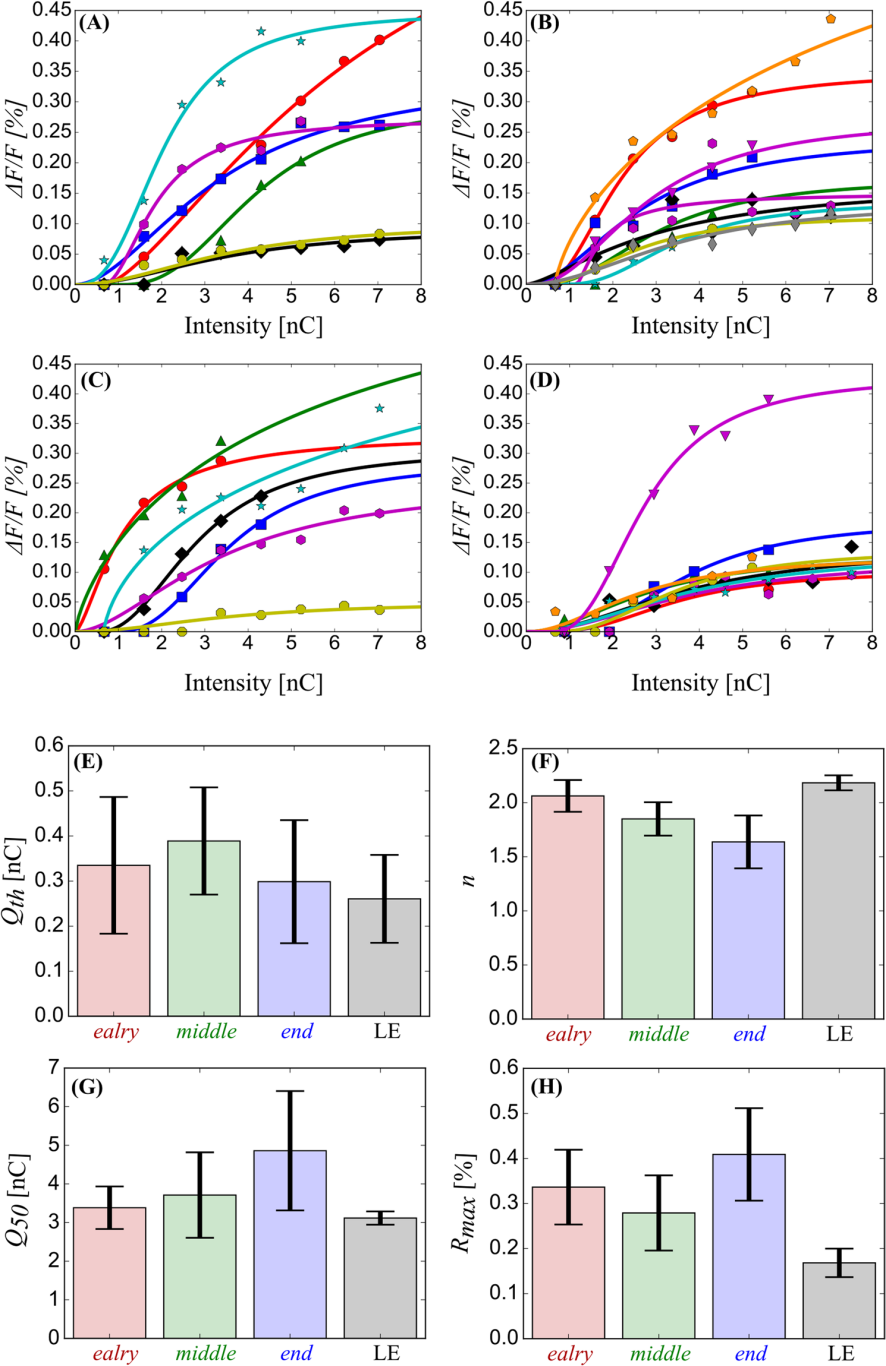
The stimulus intensity dependence of the response. (**A**–**D**) The intensity-response curves for the *early-* (**A**), *middle-* (**B**), and *end*-stage RCS (**C**), and LE (**D**) rat groups. The symbols and lines are the measured values and fitted lines using a modified Naka-Rushton function (Equation 1). There is a similar trend in all groups. (**E**–**H**) Comparisons of *Q*_*th*_ (**E**), *n* (**F**), *Q*_50_ (**G**), and *R*_*max*_ (**H**; Equation 1) in each group (mean ± SEM, *p* > 0.05).

We measured the stimulus intensity vs. the response amplitude curve of the electrically induced cortical responses in the RCS rats and the LE rats, as illustrated in Fig. 5. The current intensity changed from 5 to 40 μA and the positive peak amplitude is plotted as a function of the current intensity. As shown in Fig. 5, a discernible response appeared at an intensity of approximately 1 nC/phase (5 μA with 200 μs). The amplitude of the response increased steeply and then gradually reached a ceiling level as the intensity increased. To characterize the dependence of the peak amplitude on the stimulation intensity, the intensity-amplitude relationship was fitted using a modified Naka-Rushton function (Equation 1; see Methods). We obtained the maximum response amplitude, slope, threshold intensity, and intensity eliciting half of the maximum amplitude, which are expressed as *R*_*max*_, *n*, *Q*_*th*_, and *Q*_50_, respectively, in Equation (1) in the Methods section. An estimation was made for each sample (*n* = 7, 10, 7, and 9 for the *early*-*, middle*-, and *end*-stage RCS rats, and LE rats, respectively). As illustrated in Fig. 5(A–D), most of the fitted curves depart from the horizontal axis at approximately 1 nC/phase and are saturated at approximately 4–5 nC/phase. The signal amplitude varies in animals even within the same group, possibly due to the degree of VSD staining, even though the signal is expressed as a ratio (*ΔF/F*; see Methods). The estimated parameter values are illustrated in Fig. 5(A–H). As shown in Fig. 5(E), the threshold intensity (*Q*_*th*_) was similar in all rat groups, i.e., approximately 0.3 nC. There was no statistically significant difference between all rat groups [*F*_(3, 25)_ = 0.192, *p* = 0.901]. The slope, *n*, was approximately 2 in the *early*-stage RCS rats, and this seemed to decrease as the degeneration progressed, as shown in Fig. 5(F). However, this change in slope was not statistically significant [*F*_(3, 25)_ = 0.192, *p* = 0.901]. The slope of the *early*-stage RCS rats was similar to that of the LE rats. There was no statistically significant difference in the half-maximum intensity, *Q*_50_, between the rat groups [Fig. 5(G); *F*_(3, 25)_ = 1.95, *p* = 0.144], suggesting that the *Q*_50_ was similar for all rat groups, although the deviations were relatively large. For the saturation value of the fitted function, *R*_*max*_ was also similar between the groups [*F*_(3, 25)_ = 1.47, *p* = 0.242].

These observations again suggest that the excitability of the cortical circuit are maintained in the RCS rat throughout the retinal degeneration process and even after the total loss of retinal input.

### Spatial profile of the excited area

**Figure 6:**
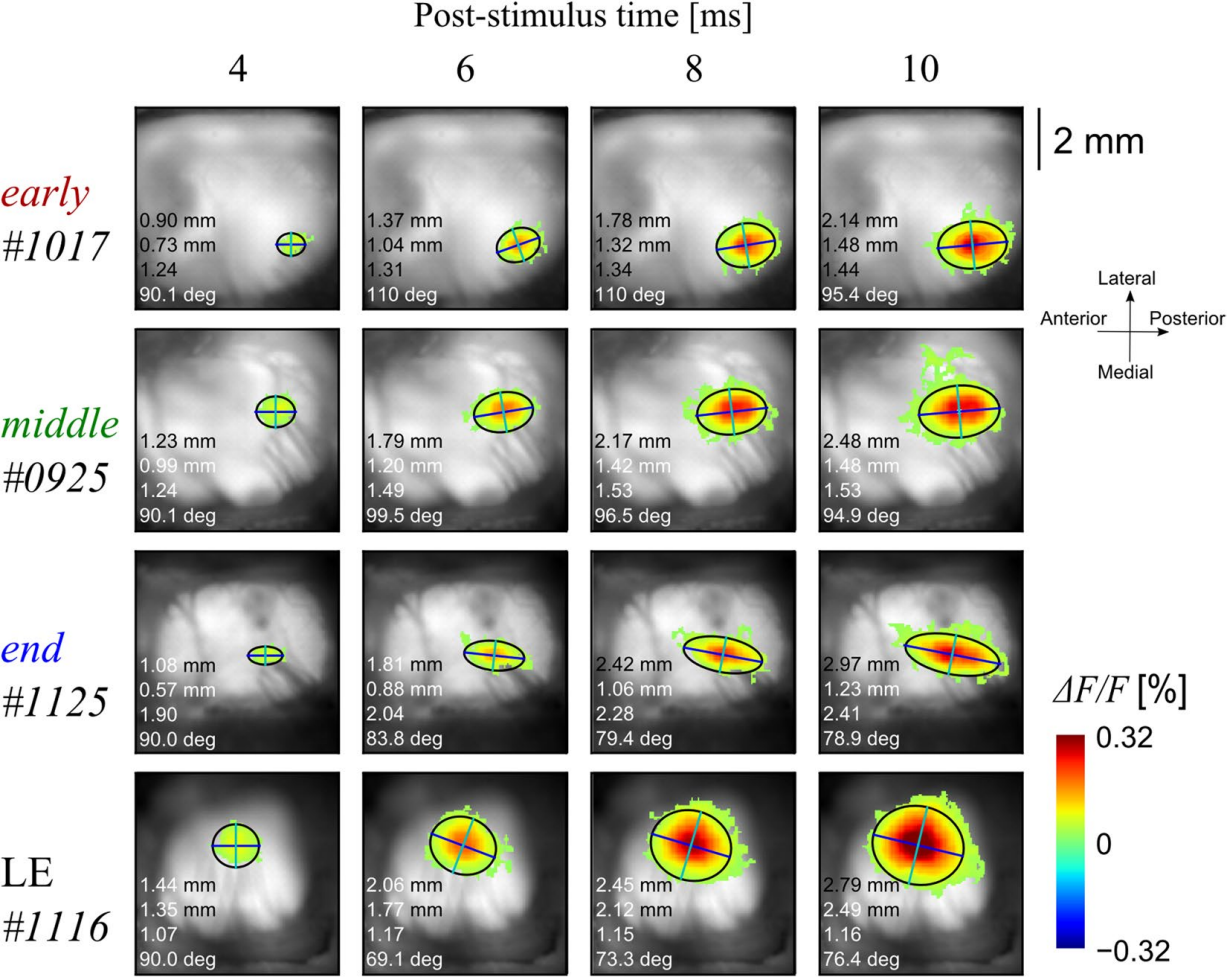
Typical examples of the Gaussian fitting map (Equation 2) at a certain time in each rat shown in Fig. 3. The fitting areas are shown as a colour scale, which was defined as the area exceeding the mean + 5 SD before the pre-stimulus timepoint at each pixel. The top numbers indicate the post-stimulus timepoint. The blue and cyan lines indicate the long and short axes estimated by fitting_,_ respectively. The black contour line indicates the outline (4*σ*) of the fitted two-dimensional Gaussian function. The numbers embedded in each panel indicate the long axis length, short axis length, aspect ratio, and angle from the top. The responses spread with time; the LE rat response spread in a circle, while the response of the older RCS rats spread elliptically.

Previous studies have indicated that the electrically activated area spreads laterally around the stimulation site via lateral synaptic connections in the rodent V1^30,31^. In the above section, we showed that progressive retinal degeneration had little effect on the excitation induced by electrical stimulation in the V1 of the RCS rat. However, it is possible that the network connections in the cortical circuit changes during the degeneration process. We investigated whether there is any progressive change in the spatial properties of the activated area at a gross scale. To quantify the differences among the groups, the activated area was fitted using a two-dimensional Gaussian function (Equation 2; see Methods). Examples are shown in Fig. 6. The four columns from the top correspond to representative *early*-, *middle*-, and *end*-stage RCS rats and an LE rat, respectively. The contours of the fitted function (see Methods) are superimposed on the VSD images obtained at 4, 6, 8, and 10 ms in the different columns. Note that the fitting area was restricted to the pixels exceeding the criterion [mean + 5 SD (standard deviations) of the pre-stimulus period at each pixel]. As shown in Fig. 6, the activated area expanded as the time after stimulation increased in all rats. The activated area appears elliptical along the anterior-posterior (AP) axis in the RCS rats and circular in the LE rat. Interestingly, the activated area appears more elongated as the degeneration progresses in these examples.

**Figure 7:**
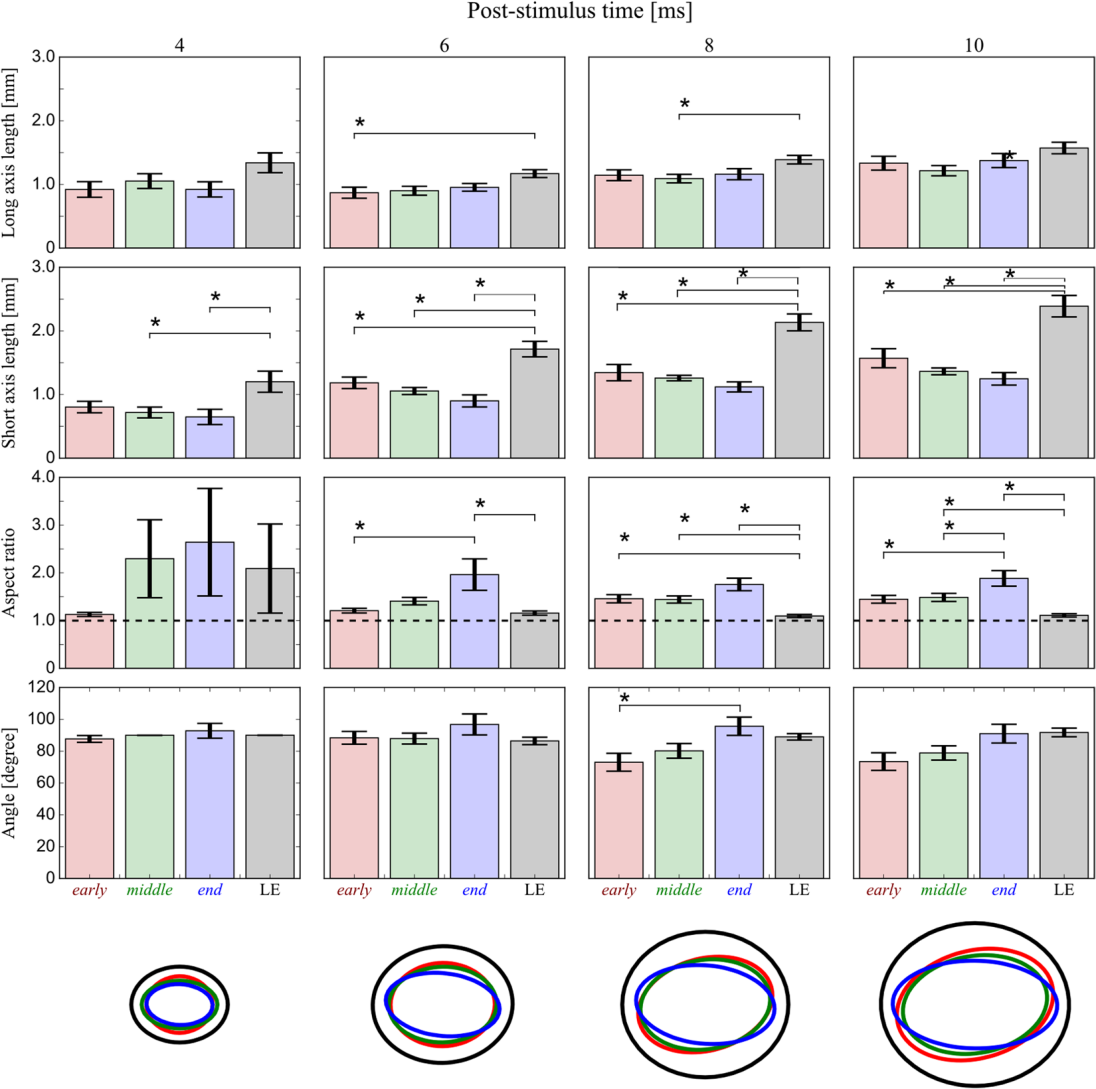
Comparison of the two-dimensional Gaussian function fitting parameters at each timepoint in each group. From the top, the long axis length, short axis length, aspect ratio, and angle are shown. The bottom row illustrates the response maps reconstructed from the averaged parameters (red, *early*; green, *middle*; blue, *end*; black, LE). The response was narrowly distributed, especially in the ML axis of the older RCS rat groups. Data are expressed as the mean ± SEM (*p < 0.05).

The aforementioned observations were confirmed to be statistically significant in the pooled data (*n* = 7, 10, 7, and 9 for the *early*-, *middle*-, *end*-stage RCS rats, and the LE rats, respectively) as shown in Fig. 7. The top and the second from the top rows illustrate the length of the long and short axes obtained in the frame at the different times. It is clear that both axes became longer as the time after the stimulation increased in all rat groups. The average length of the long axis (the top panels) was similar in all rat groups [at the 4-ms frame: *F*_(3,25)_ = 1.97, *p* = 0.130; 10 ms: *F*_(3,25)_ = 2.39, *p* = 0.089], or slightly longer in the LE rat group [6 ms: *F*_(3,25)_ = 3.41, *p* = 0.03, *early* vs. LE, *q*_(4,25)_ = 3.90, *p* = 0.047; 8 ms: *F*_(3,25)_ = 2.96, *p* = 0.049, *middle* vs. LE, *q*_(4,25)_ = 3.97, *p* = 0.042].

However, the average length of the short axis (second panels from the top) was noticeably shorter in the RCS rats, especially in the older animals [4 ms: *F*_(3,25)_ = 3.78, *p* = 0.021, *middle* vs. LE, *q*_(4,25)_ = 3.98, *p* = 0.041, *end* vs. LE, *q*_(4,25)_ = 4.15, *p* = 0.031; 6 ms: *F*_(3,25)_ = 13.1, *p* = 1.39 × 10^−5^, *early* vs. LE, *q*_(4,25)_ = 5.22, *p* = 0.005, *middle* vs. LE, *q*_(4,25)_ = 7.11, *p* = 0.001, *end* vs. LE, *q*_(4,25)_ = 8.01, *p* = 0.001; 8 ms: *F*_(3,25)_ = 19.1, *p* = 4.93 × 10^−7^, *early* vs. LE, *q*_(4,25)_ = 7.23, *p* = 0.001, *middle* vs. LE, *q*_(4,25)_ = 8.78, *p* = 0.001, *end* vs. LE, *q*_(4,25)_ = 9.30, *p* = 0.001; 10 ms: *F*_(3,25)_ = 16.0, *p* = 2.47 × 10^−6^, *early* vs. LE, *q*_(4,25)_ = 6.05, *p* = 0.001, *middle* vs. LE, *q*_(4,25)_ = 8.32, *p* = 0.001, *end* vs. LE, *q*_(4,25)_ = 8.45, *p* = 0.001].

Consequently, the aspect ratios (third panels from the top) of the RCS rat groups were greater than that of the LE rat at all frames except for the 4-ms frame [4 ms: *F*_(3,25)_ = 0.436, *p* = 0.728; 6 ms: *F*_(3,25)_ = 4.67, *p* = 0.009, *early* vs. LE, *q*_(4,25)_ = 4.32, *p* = 0.023, *end* vs. LE, *q*_(4,25)_ = 4.89, *p* = 0.008; 8 ms: *F*_(3,25)_ = 9.12, *p* = 2.07 × 10^−4^, *early* vs. LE, *q*_(4,25)_ = 4.01, *p* = 0.039, *middle* vs. LE, *q*_(4,25)_ = 4.21, *p* = 0.028, *end* vs. LE, *q*_(4,25)_ = 7.33, *p* = 0.001; 10 ms: *F*_(3,25)_ = 9.37, *p* = 1.71 × 10^−4^, *early* vs. *end*, *q*_(4,25)_ = 3.98, *p* = 0.041, *middle* vs. *end*, *q*_(4,25)_ = 3.93, *p* = 0.044, *middle* vs. LE, *q*_(4,25)_ = 3.99, *p* = 0.040, *end* vs. LE, *q*_(4,25)_ = 7.48, *p* = 0.001]. Note that some samples exhibited very small axis length values at 4 ms, resulting in an extremely high aspect ratio in the *middle*- and *end*-stage RCS rat groups, and in the LE rat group.

The panels in the 4th row show the angle of elongation. The V1 circuit of the LE rat is rather isotropic; the aspect ratio was almost 1, so the angle of 90° probably just represents an initial value of the fitting procedure. The averaged angle was between 73° and 97° in all RCS rat groups. These values were similar among the groups at early timepoints [4 ms: *F*_(3,25)_ = 0.691, *p* = 0.564; 6 ms: *F*_(3,25)_ = 1.04, *p* = 0.385]. At later timepoints, younger animals exhibited slightly smaller angle values [8 ms: *F*_(3,25)_ = 3.71, *p* = 0.023, *early* vs. *end*, *q*_(4,25)_ = 4.30, *p* = 0.024; 10 ms: *F*_(3,25)_ = 3.20, *p* = 0.038; no significant difference was observed in the post hoc tests], meaning that the elongation was slightly biased towards the medioanterior-lateroposterior axis by 17° and 9.8° for the *early* and *middle* RCS rats, respectively.

These observations suggest that the V1 circuit of the RCS rat is anisotropic, orienting along the AP axis (bottom row in Fig. 7). In other words, the lateral synaptic connections in the V1 circuit of the RCS rat may somehow degenerate, especially in the medial-lateral (ML) direction over the course of retinal input loss. At the same time, the medioanterior and/or lateroposterior sections of the V1 circuit may be relatively maintained in younger RCS rats.

## Discussion

The RCS rat is a commonly used animal model of blindness caused by retinal degeneration^3–5,8–10,33–35^. In the present study, we examined the spatiotemporal properties of cortical responses induced by optical and electrical stimuli over the course of retinal degeneration in the RCS rat and compared these properties with those of the LE rat using VSD imaging *in vivo*. VSD imaging enables an estimation of the spatiotemporal changes of PSPs in the local neuronal population^26–28^, without any artefacts from electrical stimulation. In addition, the spatiotemporal resolution of the VSD signal is higher than that of LFPs.

The cortical response to optical stimulation exhibited a longer response latency and a slower time-course as degeneration progressed in the RCS rat (Figs 1 and 2). Previous studies have reported that the response latency to optical stimuli in the cortical circuit of 4-, 7-, and 14-week-old RCS rats showed similar results, as measured with the LFP and MUA^3^. These rats are equivalent to the *early*- and *middle*-stage RCS rats in the current study. The response latency progressively increased in the RCS rat with age. The response latency in the control group was within 25–35 ms, while that of the 4-, 7-, and 14-week-old RCS rats was within 35–50, 50–65, and 65–80 ms, respectively, judged by eye from the figures in ref.^3^. This age-related degradation of the light-induced cortical response, with regard to the response latency and the rising time, is thought to be inherited in the retina and not due to changes in the visual pathway downstream of the retina, such as the LGN or optic radiation, since a longer response latency was also reported in the retinal^6,7^, as well as in the SC^4,8^. In addition, the response time-course, measured with the decaying time tended to become slower as degeneration progressed in the RCS rat in the present study. Pinilla *et al*. reported similar results, in which the decaying time also seemed to be prolonged on the ERG^6^ in response to a flash of light. Thus, the time-course changes in the cortical response to a flash of light stimulus in the RCS rat are considered to reflect the retinal activity changes during the process of degeneration.

The RCS rat showed a long response latency and slow time-course even from a very early stage (3 weeks) of retinal degeneration compared to those observed in the LE rat. These differences in the response latency and time-course may affect visually guided behaviour, such as maze exploration, and the behavioural response to light stimuli, such as flickering lights and frequency-modulated sinusoidal gratings, in the RCS rat^36–38^. However, such a prolonged response latency was not observed in the electrically induced response in the RCS rat. The cortical circuit was excited promptly even in the *end*-stage RCS rat that exhibited no light-induced responses, and there was no significant difference between the RCS rat and the LE rat in terms of excitation [Fig. 4(B) and (C)]. Furthermore, we revealed that there was no discernible difference in the dependence of electrically induced excitation on intensity between the RCS rat groups and the LE rat group (Fig. 5). Data from both the RCS rats and the LE rats showed similar current threshold intensities (*Q*_*th*_), intensities that elicited half of the maximum response (*Q*_50_), and slopes (*n*) when fitted with a modified Naka-Rushton function. The estimated *Q*_*th*_ (less than 0.8 nC/phase) and *Q*_50_ (in a range between 2.5–3.0 nC/phase) are comparable to previous studies in other animal species and other cortical areas. For example, Stoney *et al*. performed extracellular recordings of responses to intracortical microstimulation in the pericruciate cortex of cats, and then elucidated the threshold current required to activate the synaptic excitation, which was approximately 1 nC/phase^39^. Butovas and Schwarz reported that at least 0.8 nC/phase of an intracortical microstimulation to the somatosensory cortex of rats could induce neural activity^40^. In addition, according to previous studies of intracortical microstimulation in the visual^41,42^, somatosensory^43^, and auditory cortices^44^, a similar or slightly higher range of stimulation intensities were shown to elicit the behavioural responses.

These results strongly suggest that the excitability in the cortical circuit maintain their activity in the RCS rats through the *end*-stage of disease and are hardly affected by the gradual loss of retinal input. The present study indicates that the RCS rat is as susceptible as normally sighted rats with regard to electrical stimulation applied directly to the visual cortex, and therefore can be used for behavioural experiments to evaluate the performance of cortical prosthetics.

**Figure 8:**
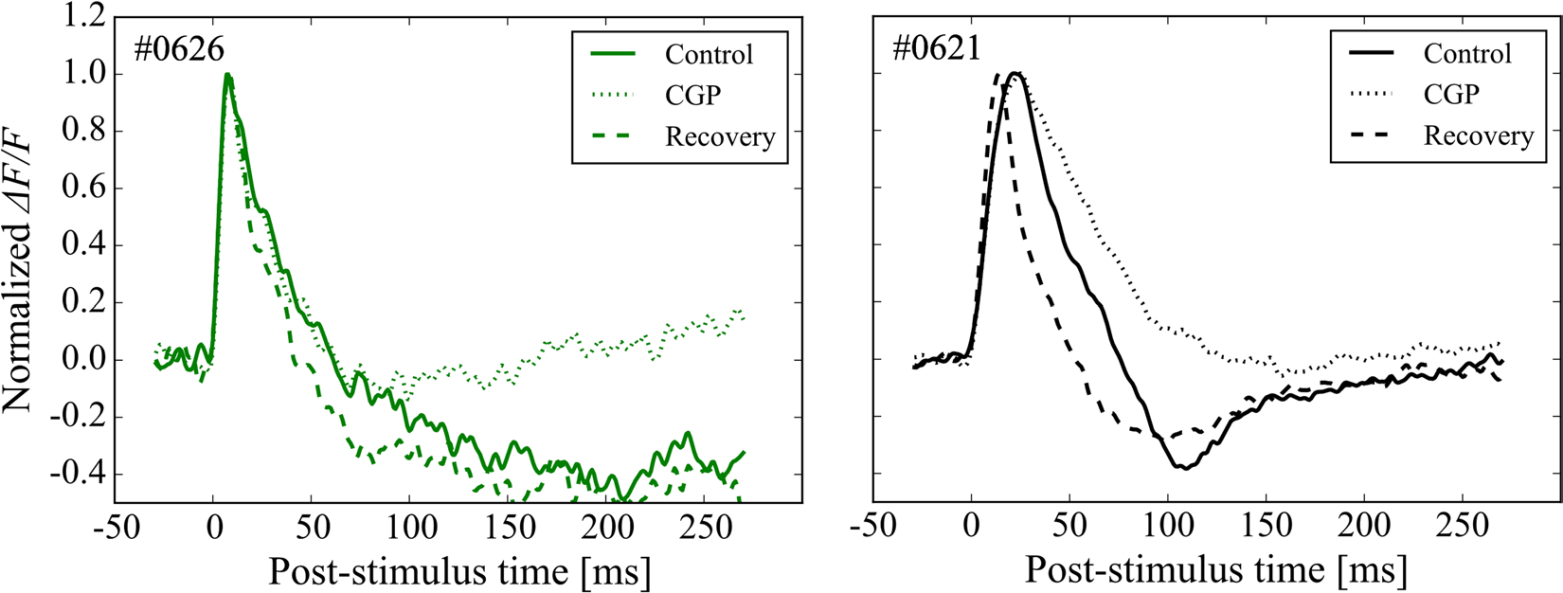
GABA-mediated inhibition of the electrically induced cortical response. The normalized time-courses at the stimulation points are shown for the *middle*-stage RCS (left) and the LE rat (right). In each figure, the solid, dashed, and dotted lines indicate the control, CGP application, and recovery condition, respectively. Overall, with CGP 46381 application, the VSD undershoot responses decreased [trough magnitude relative to the peak value in control/CGP/recovery conditions = 0.51/0.10/0.52 for RCS, and 0.41/0.05/0.30 for LE]. The peak-to-trough times in the CGP conditions were longer than the control and recovery conditions in the LE rats (control/CGP/recovery = 87/133/86 ms). On the other hand, CGP application shortened the peak-to-trough times for the RCS rats (control/CGP/recovery = 202/92/147 ms).

In contrast, the response time-course of the falling phase in the electrically induced response appeared to be different between the LE and RCS rats [Fig. 4(B) and (D)]. An obvious difference between the LE and RCS rats was the prominent undershoot response that was distinct in the LE rats (eight of nine animals). The undershoot response was not observed, or was weak, in the RCS rats. Moreover, the time-course was much slower in the RCS rats than in the LE rats [Fig. 4(D)]. The difference in the undershoot response was also observed in the light-induced response [Fig. 2(A), for example]. The time-course of undershoot resembles the electrically induced cortical response of mice measured with *in vivo* VSD imaging that was suppressed with the GABAergic synaptic transmission blockers gabazine and bicuculline^30^. A similar long-lasting response was observed in the intracellular response to an extracellularly applied electrical shock obtained in slice preparations of rats, and these undershoot responses were identified as the GABA_B_-mediated IPSPs^32^. From these previous experiments it is likely that the undershoot observed in the present study reflects a GABA_B_-mediated population IPSP. Therefore, we performed pharmacological experiments to examine the effect of a GABA_B_ receptor antagonist, CGP 46381, on the electrically induced responses of the LE rat and the *middle*-stage RCS rat that exhibited the undershoot response. As shown in Fig. 8, CGP reversibly blocked the long-lasting undershoot responses. We performed this pharmacological experiment on two LE rats and two *middle*-stage RCS rats and obtained similar results in all animals. Although these pharmacological experiments are preliminary, the results are consistent with previous observations^30,32^ and suggest that the suppressive activity in this cortical circuit are reduced in the RCS rat, while the properties of the excitability are maintained.

Finally, in the current study we showed that intracortical electrical microstimulation elicited an almost circular response, spreading more than 1.0 mm in radius, in the LE rat group. In contrast, the RCS rat groups exhibited a response area that was more elliptical in shape, oriented in the AP direction. More importantly, the spread of excitation, especially in the ML direction, was suppressed as the degeneration of the retina progressed, suggesting that the synaptic connections in the ML direction in the V1 circuit of RCS rats are susceptible to visual input loss during retinal degeneration. Further experiments are necessary to elucidate the effects of retinal degeneration on the cortical circuit structure in the RCS rat.

## Methods

### Animals

All experiments were approved by the Institutional Animal Care and Use Committee of the Graduate School of Engineering, Osaka University and were conducted in accordance with the Guiding Principles for the Care and Use of Animals in the Field of Physiological Sciences of the Physiological Society of Japan and the Guidelines for Animal Experiments of Osaka University. Animals were maintained in a 12:12-h light/dark cycle environment. All surgeries and recordings were performed under urethane anaesthesia, and all efforts were made to minimize suffering. The animals were euthanized by over-inhalation of halothane after each experiment.

The RCS rats (female rats; CLEA Japan, Inc., Tokyo, Japan) were divided into three groups according to their age and are referred to as *early* (3–5-weeks-old, *n* = 5), *middle* (9–13-weeks-old, *n* = 7), and *end* (88–100-weeks-old, *n* = 5) stages to consider the retinal degeneration-related differences in visual function. As a control, we used LE rats (8–12 weeks old; SLC Japan, Inc., Ibaraki, Japan; n = 5). The responses were compared between the groups. In experiments using electrical stimulation, we used multi-channel electrodes (see Electrical Stimulation section), and the data for each channel were handled as individual samples (*n* for *early*-, *middle*-, and *end*-stage RCS rats and LE rats = 7, 10, 7, and 9, respectively).

### Imaging

#### Surgery

All surgical and recording procedures conducted in the current study were based on our previous studies^30,45^. We describe the procedures briefly here. Anaesthesia was induced with an intraperitoneal (i.p.) injection of urethane (25%, 5 μl/g body weight; Tokyo Chemical Industry Co., LTD, Tokyo, Japan) and a further small dose was added when needed. After that, atropine (0.05 μg/ml, 0.2 ml, i.p.; Sigma-Aldrich Inc., MO, USA) was administered to inhibit mucus secretion. Dexamethasone was administered by intramuscular injection (0.1 mg/ml, 0.2 ml; Sigma-Aldrich Inc.) to inhibit inflammation. Subsequently, the animal was placed in the stereotaxic apparatus (SR-10R-HT; NARISHIGE, Tokyo, Japan), and the ear bar was fixed onto the animal. Body temperature was maintained at 37.3 °C using a heating pad. Oxygen was applied directly into the animal’s nose. Electrocardiogram (ECG) data were measured during the experiment. Before the craniotomy, a local anaesthetic was injected under the skin of animal’s head. To prevent VSD from flowing out of the exposed cortex, we attached an enclosure (8 × 7 mm) that was made with a 3D printer (BS01; Bonsai Lab Inc., Tokyo, Japan) onto the bone above the visual cortex. A craniotomy was performed on the right posterior bone to expose the visual cortex (AP, 1–8 mm from bregma; ML, 1–6 mm from the sagittal suture; ref.^46^). After peeling the dura matter back, staining was performed using a bath-application of VSD (RH1691; Optical Imaging, Rehovot, Israel) for 90 minutes, and washing was carried out using artificial cerebrospinal fluid (ACSF; 125 mM NaCl, 2.5 mM KCl, 0.9 mM NaH_2_PO_4_, 5 mM Na_2_HPO_4_, 1.2 mM CaCl_2_, 1.0 mM MgCl_2_, and 2.5 mM D-Glucose).

#### Recording

To record the changes in dye fluorescence along with neural activities *in vivo*, we used epifluorescence microscopy. A halogen lamp was used as the excitation light (MHF-G150LR; MORITEX Inc., Saitama, Japan). The excitation light was controlled by a shutter to prevent dye bleaching. The emitted fluorescence passed through a dichroic mirror and barrier filter and was acquired by a CMOS-camera-based imaging system (MiCAM ULTIMA; BrainVision Inc., Tokyo, Japan), which has a 100 × 100-pixel resolution and 1-kHz frame rate. The size of the sensor array was 10 × 10 mm, which could acquire a 6.25 × 6.25 mm area of the visual cortex with a 1.60 magnification (62.5 × 62.5 μm per pixel). This system enabled the response rise time, defined as the pixel which had the shortest response latency, to be found (see Data Analysis section). Acquisitions were triggered by the R component of the ECG and were taken with stimulation and without stimulation alternately at approximately 10-s intervals. The data without stimulation were subtracted from that with stimulation. This procedure permitted a reduction of the artefacts with vascular pulsation and dye bleaching^47^.

### Stimulation

#### Light stimulation

In each experiment, a light-evoked response was recorded to investigate the neural dynamics of brightness vision. As a light stimulus, a 525-nm-wavelength light-emitting diode (LED) was used (STS-DA1–2348; NICHIA Inc., Tokushima, Japan). The LED was placed in front of the left eye of the animal with a cover tube that prevented the light from entering the recording area. The photon densities used were 1132 or 1442 photons/(μm^2^s) with a 3.03 or 4.63 mA current supply to the LED, respectively. The pulse duration was 100 ms.

#### Electrical stimulation

Electrical stimuli were delivered through a 4-channel electrode array (MicroProbe Inc., MD, USA) with activated-iridium tips. An approximately 100-μm long and 30-μm wide area of the electrode was exposed. The tip diameter was 1–2 μm. The distance between the electrodes was approximately 300 μm^48^. The electrodes were inserted into the V1 area, which was confirmed by the light-stimulation response. For the *end*-stage RCS rat group, we confirmed the V1 area using a rat brain atlas^46^. The tip of the electrode was oriented 600 μm perpendicular below the dura (layer IV; ref.^49^). The electrode impedance was 18.3–40.0 kΩ at 1 kHz. The cathodic-first biphasic current pulses (intensity 5–40 µA/phase, duration 200 µs, no interphase interval) were delivered with an isolated current generator (STG2008; Multi Channel Systems, Reutlingen, Germany) for the RCS rat groups, and with a stimulation chip that we previously developed for the LE rat group^48^. The stimulus intensity was expressed as a charge transfer, which was calculated by a measured waveform (not shown). The charge transfer used in the present study was 0.67–7.04 nC/phase and 0.88–7.52 nC/phase for the RCS rat groups and the LE rat group, respectively.

#### Pharmacology

To investigate the mechanism underlying in the undershoot signal shown in the VSD signals, the GABA_B_ antagonist CGP 46381 (100 or 200 μM in ACSF; Tocris, Bristol, UK) was applied to the visual cortex of the *middle*-stage RCS rats and the LE rats (*n* = 2 each). In this study, we focused on the effect of the GABA_B_ receptor, which relates to the slower inhibitory response (approximately 100–200 ms) caused by electrical stimulation in the rat visual cortex^50^. We sequentially made the recordings with ACSF (“control”), with CGP 46381 filling into the recording enclosure (“CGP”), and with ACSF again (“recovery”). To reduce noise, the data were averaged 40–161 times for each condition. The charge transfer was within 5.22–7.04 nC/phase.

### Data Analysis

Data analysis was performed with custom-written procedures in Python (Python Software Foundation, DE, USA) and Matlab (The MathWorks, MA, USA). In each pixel of an acquired image sequence, the mean value for 30 or 50 ms before stimulation was calculated (*F*_0_), and the difference between the fluorescence intensity of each frame and *F*_0_ was calculated (*ΔF*). By dividing *ΔF* by *F*_0_, the fluorescent relative change (*ΔF/F*) was calculated. The *ΔF/F* was proportional to the relative membrane potential^47,51^. To reduce noise, the stimulus was repeated 8–32 times with the same intensity, and the acquired *ΔF/F* image sequences were averaged. A Gaussian spatial filter and a third-order Bessel temporal filter (cut-off frequency = 50 Hz) were applied to all of the data before quantitative analysis. For the flashing-light stimulation and the spatial property analysis following the electrical stimulation, a 7 × 7-pixel Gaussian spatial filter (σ ~ 1.4 pixels) was used. For the temporal properties and stimulus dependence analysis, a 15 × 15-pixel Gaussian filter (σ ~ 2 pixels) was used.

To obtain the time-course of the response to the flashing light, we defined an 11 × 11-pixel region, which was centred on a pixel having the shortest latency (see below) and within the V1 area based on the rat brain atlas^46,52,53^, and we averaged the values of all pixels in the region. For the quantitative analysis, the following parameters were assessed: response latency, rising time, decaying time, and peak-to-trough time. The response latency was calculated as follows: the pre-stimulus signals (from 30 ms before the stimulation) were fitted with a straight line by means of the least squares method (command “minimize” with sequential least squares programming method in SciPy, Python). The mean of the residuals between the data and the fitted line was calculated during the pre-stimulation period. The response latency time was defined as the earliest post-stimulation timepoint at which the difference between the values of the recorded signal and the extrapolation of the fitted line exceeded six times the mean residual value. The rising time is the time from the response latency to the maximum (positive) response peak. The decaying time is the time from the maximum response peak to half of the maximum response. The peak-to-trough time is the time from the maximum response peak to the minimum (negative) response trough. When calculating the peak-to-trough time, samples with no undershoot were excluded. We defined undershoot as a negative response after a maximum peak, which lasted longer than 50 ms and less than 250 ms. The minimum peak was identified in the undershoot period. We confirmed that these temporal parameters depend less on the centre positioning (Fig. S1).

For numerical assessments, in addition to some temporal parameters, such as the light-stimulation data (response latency and peak-to-trough time), the stimulation-intensity dependence and spatial properties of electrical stimulation-induced responses were investigated. The relationship between the stimulus intensities and *ΔF/F* responses at the stimulus point at the peak time was investigated (intensity-response curve), which was fitted using a modified Naka-Rushton function^54,55^:

1 $$\begin{array}{rcl}\frac{\Delta F}{F}({Q}_{diff}) & = & \frac{{R}_{\max }{{Q}_{diff}}^{n}}{{{Q}_{diff}}^{n}+{({Q}_{50}-{Q}_{th})}^{n}}\\ {Q}_{diff} & = & \{\begin{array}{c}Q-{Q}_{th}\quad if\quad Q-{Q}_{th} > 0\\ 0\quad \quad \quad \,\,\,otherwise\end{array},\end{array}$$

where *R*_*max*_, *n*, *Q*_50_, and *Q*_*th*_ indicate the maximum response, slope, intensity eliciting a half-maximum response, and threshold intensity, respectively. The fitted parameters were compared between the rat groups. To investigate the spatial extent of the response evoked by the electrical stimulation, the response maps at certain times were fitted using a two-dimensional Gaussian function:

2 $$\begin{array}{rcl}\frac{\Delta F}{F}(v,w) & = & A\exp \{-(\frac{{v}^{2}}{2{{\sigma }_{v}}^{2}}+\frac{{w}^{2}}{2{{\sigma }_{w}}^{2}})\}\\ v(x,y) & = & (x-{x}_{0})\cos \,\theta +(y-{y}_{0})\sin \,\theta \\ w(x,y) & = & -(x-{x}_{0})\sin \,\theta +(y-{y}_{0})\cos \,\theta ,\end{array}$$

where *A*, *σ*, *x*_0_, and *y*_0_ represent the amplitude, spatial extent, centre position on the AP axis, and centre position on the ML axis of the response map, respectively. *v* and *w* indicate the long and short axes of the map, respectively. *θ* is the angle of the long axis to the ML axis, where positive values indicate rotations from the lateral to the posterior axis. *θ* (angle), 4*σ*_*v*_ (long axis length), 4*σ*_*w*_ (short axis length), and *σ*_*v*_/*σ*_*w*_ (aspect ratio) were compared between the rat groups. The fitting was performed using pixels with a response exceeding the mean + 5 SD of the pre-stimulation level only to eliminate artefacts caused by vascular pulsation and lack of staining. Response maps with a maximum charge transfer of 3.37–7.52 nC/phase were used for the fitting.

To compare the responses between the RCS rat groups and the LE rat group, statistical procedures were conducted. A one-way analysis of variance (ANOVA) was carried out followed by a post hoc Tukey-Kramer method (Figs 2, 4, 5 and 7). The significance level was set at *α* = 0.05. The statistical results of the post hoc Tukey-Kramer method for non-significant cases are omitted for better readability.

## Electronic supplementary material

Supplementary Figure
