# Supplementary material for: Age-related changes in the spatiotemporal responses to electrical stimulation in the visual cortex of rats with progressive vision loss
Source: Sci Rep. 2017 Oct 26;7:14165. doi: 10.1038/s41598-017-14303-1 (PMC5658441; doi:10.1038/s41598-017-14303-1)
Supplement: Supplementary file 1 — Supplementary Figure [file 41598_2017_14303_MOESM1_ESM.pdf]

Age-related changes in the spatiotemporal responses to electrical stimulation in the visual cortex of rats with progressive vision loss

Soshi Miyamoto, Naofumi Suematsu, Yuichi Umehira, Yuki Hayashida, Tetsuya Yagi*

Biosystems and Devices Area, Division of Electrical, Electronic and Information Engineering, Department of Electronic Engineering, Graduate School of Engineering, Osaka University

2-1 Yamadaoka, Suita, Osaka 565-0871, Japan

*Corresponding author

Contact information:

Tetsuya Yagi Ph.D.

Biosystems and Devices Area, Division of Electrical, Electronic and Information Engineering, Department of Electronic Engineering, Graduate School of Engineering, Osaka University

2-1 Yamadaoka, Suita, Osaka 565-0871, Japan

Tel: +81-6-6879-7786

Fax: +81-6-6879-7784

Email: yagi@eei.eng.osaka-u.ac.jp

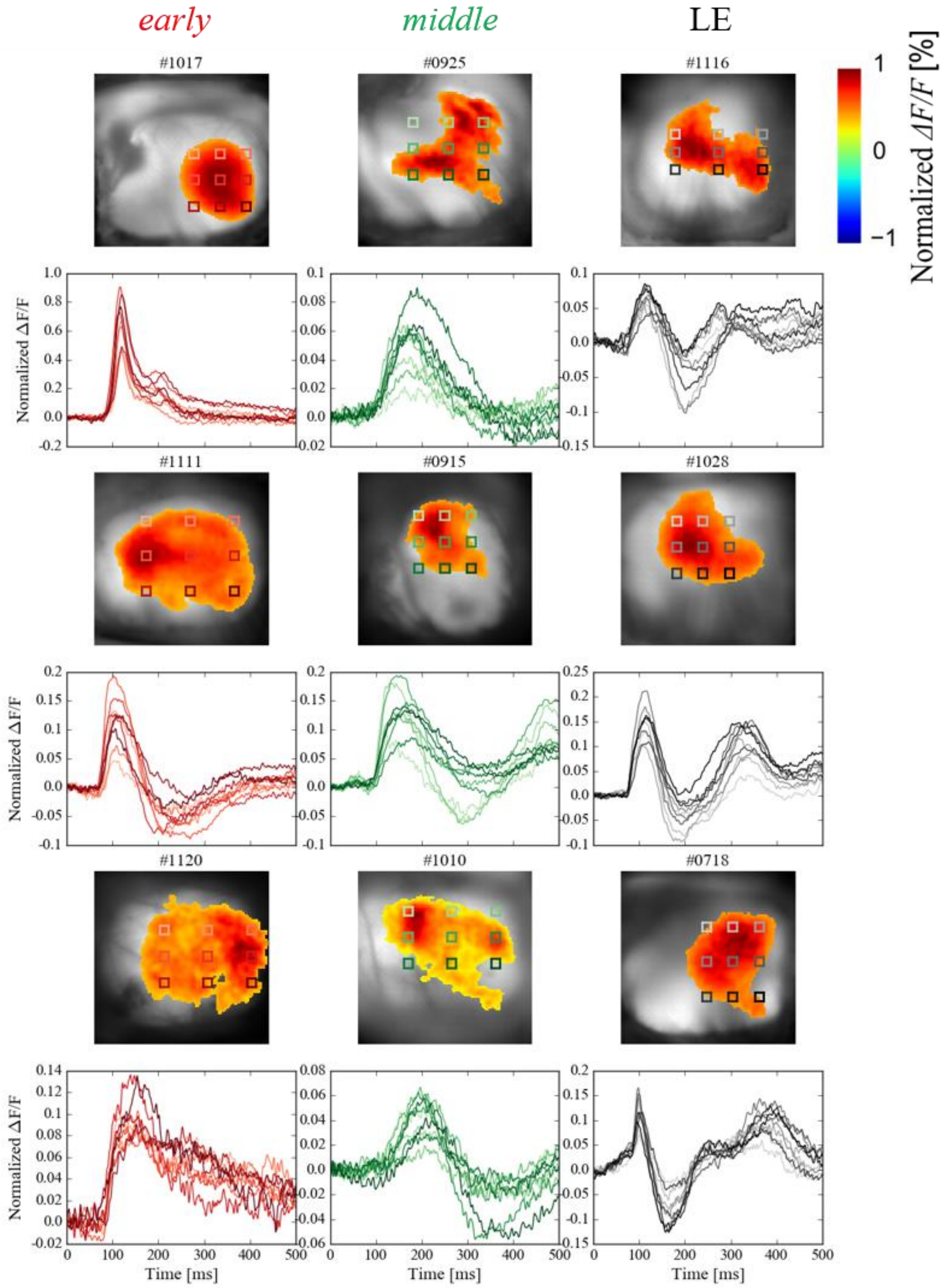

early
middle
LE
#1017
#0925
#1116
1
0
−1
Normalized ΔF/F [%]
Normalized ΔF/F
#1111
#0915
#1028
Normalized ΔF/F
#1120
#1010
#0718
Normalized ΔF/F
Time [ms]
Time [ms]
Time [ms]

Supplementary Figure 1

Time-courses at some points for three typical examples in each group.

Each colour-scaled image shows the area exceeding the half of peak amplitude at a pixel having the maximum response. Nine 5×5-pixel regions (coloured squares) were chosen within the approximately colour-scaled area, and the time-courses averaged in the regions are plotted (line colours correspond to ones of the squares). Red, green, and black lines indicate the *early*, *middle*, and LE rats, respectively. Intra-individual differences were much smaller than inter-individual ones.
